# 2-(4*H*-1,3-Benzoxazin-2-yl)phenol

**DOI:** 10.1107/S1600536809047631

**Published:** 2009-11-18

**Authors:** Christiane Fernandes, Adolfo Horn Jr, R. Alan Howie, Jan Schripsma, James L. Wardell, Edward R. T. Tiekink

**Affiliations:** a Laboratório de Ciências Químicas, Universidade Estadual do Norte, Fluminense-UENF, 28013-602, Campos dos Goytacazes, RJ, Brazil; b Department of Chemistry, University of Aberdeen, Old Aberdeen, AB15 5NY, Scotland; c Centro de Desenvolvimento Tecnológico em Saúde (CDTS), Fundação Oswaldo Cruz (FIOCRUZ), Casa Amarela, Campus de Manguinhos, Av. Brasil 4365, 21040-900, Rio de Janeiro, RJ, Brazil; d Department of Chemistry, University of Malaya, 50603 Kuala Lumpur, Malaysia

## Abstract

The title compound, C_14_H_11_NO_2_, features an essentially planar mol­ecule, the r.m.s. deviation for the 17 non-H atoms being 0.035 Å. This conformation is stabilized by an intra­molecular O—H⋯N hydrogen bond that results in the formation of an *S*(6) ring. In the crystal structure, methyl­ene–hydr­oxy C—H⋯O contacts result in a supra­molecular chain aligned along the *b* axis.

## Related literature

For general background to the synthesis, see: Hunter & Sims (1972*a* ▶,*b* ▶); Corey, & Kühnle (1997 ▶); Larter *et al.* (1998 ▶); Chou *et al.* (2004 ▶); Fernandes *et al.* (2007 ▶). For the reactions of 2-hydroxy­benzaldehyde derivatives, see; Kitan *et al.* (1990 ▶); Kanakarajan *et al.* (1975 ▶); Meier *et al.* (1979 ▶); Beer *et al.* (1948 ▶).
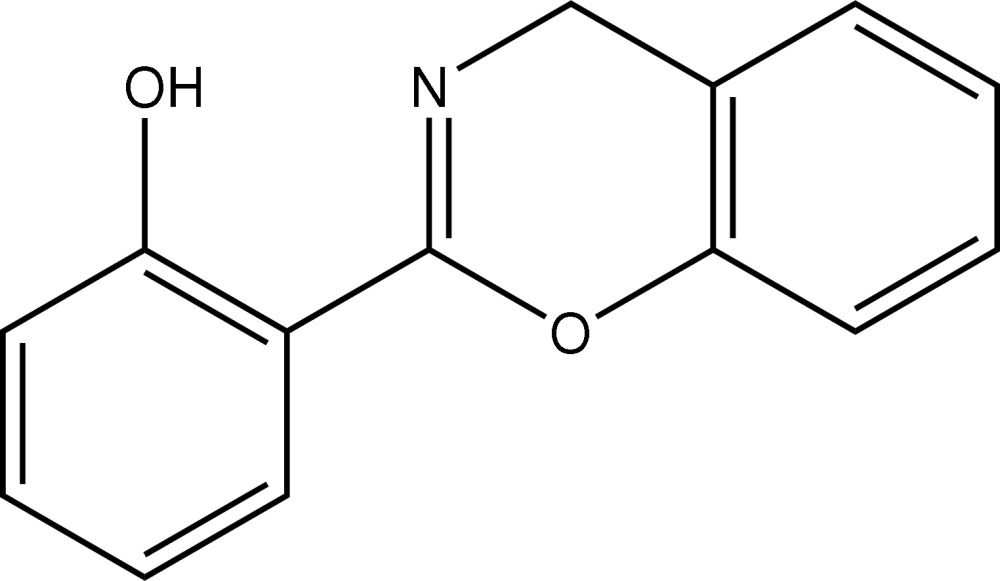


## Experimental

### 

#### Crystal data

C_14_H_11_NO_2_
*M* *_r_* = 225.24
Monoclinic, 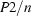

*a* = 14.1148 (8) Å
*b* = 5.1725 (2) Å
*c* = 15.8813 (8) Å
β = 115.032 (2)°
*V* = 1050.57 (9) Å^3^
*Z* = 4
Mo *K*α radiation
μ = 0.10 mm^−1^
*T* = 120 K
0.55 × 0.08 × 0.08 mm

#### Data collection

Bruker–Nonius 95mm CCD camera on κ-goniostat diffractometer
Absorption correction: multi-scan (*SADABS*; Sheldrick, 2003 ▶) *T*_min_ = 0.949, *T*_max_ = 0.992
10487 measured reflections
1841 independent reflections
1479 reflections with *I* > 2σ(*I*)
*R*_int_ = 0.066

#### Refinement

*R*[*F* ^2^ > 2σ(*F* ^2^)] = 0.048
*wR*(*F* ^2^) = 0.122
*S* = 1.07
1841 reflections
158 parameters
1 restraint
H-atom parameters constrained
Δρ_max_ = 0.32 e Å^−3^
Δρ_min_ = −0.32 e Å^−3^

### 

Data collection: *COLLECT* (Hooft, 1998 ▶); cell refinement: *DENZO* (Otwinowski & Minor, 1997 ▶) and *COLLECT*; data reduction: *DENZO* and *COLLECT*; program(s) used to solve structure: *SHELXS97* (Sheldrick, 2008 ▶); program(s) used to refine structure: *SHELXL97* (Sheldrick, 2008 ▶); molecular graphics: *DIAMOND* (Brandenburg, 2006 ▶); software used to prepare material for publication: *publCIF* (Westrip, 2009 ▶).

## Supplementary Material

Crystal structure: contains datablocks global, I. DOI: 10.1107/S1600536809047631/lh2952sup1.cif

Structure factors: contains datablocks I. DOI: 10.1107/S1600536809047631/lh2952Isup2.hkl

Additional supplementary materials:  crystallographic information; 3D view; checkCIF report

## Figures and Tables

**Table 1:** Hydrogen-bond geometry (Å, °)

*D*—H⋯*A*	*D*—H	H⋯*A*	*D*⋯*A*	*D*—H⋯*A*
O1—H1*O*⋯N1	0.84	1.78	2.5586 (16)	154
C14—H14*A*⋯O1^i^	0.99	2.45	3.3551 (19)	151

Symmetry code: (i) 
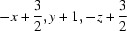
.

**Fig. 1.**
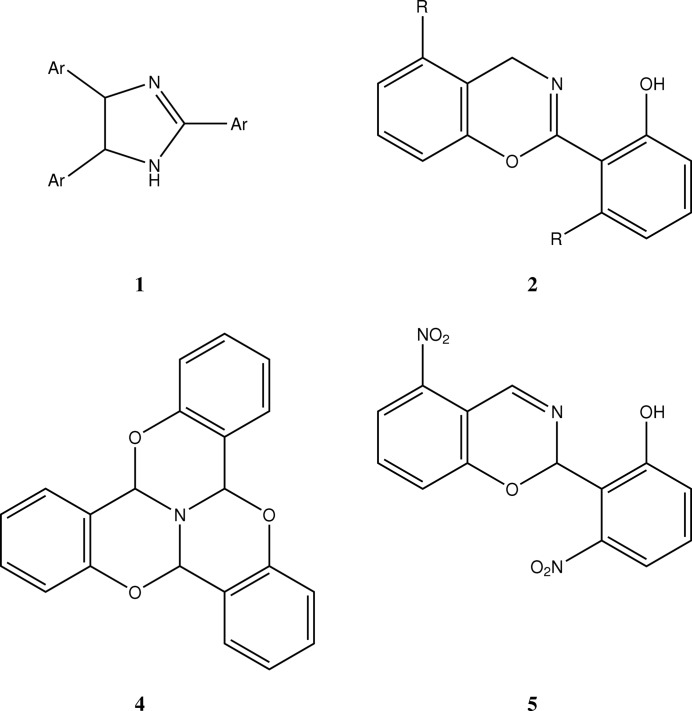
Reaction products.

**Fig. 2.**
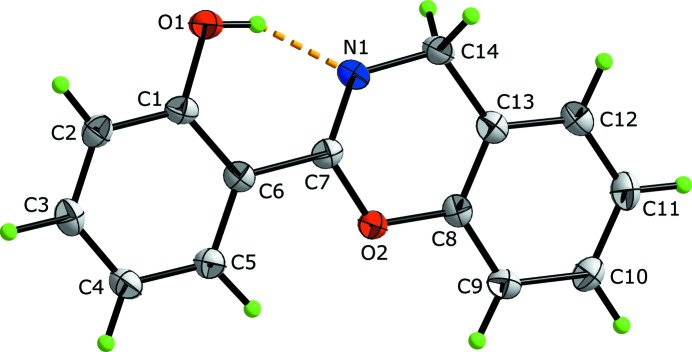
Molecular structure of (I) showing the intramolecular O–H···N hydrogen bonding (orange dashed lines), the atom-labelling scheme, and displacement ellipsoids at the 50% probability level.

**Fig. 3.**
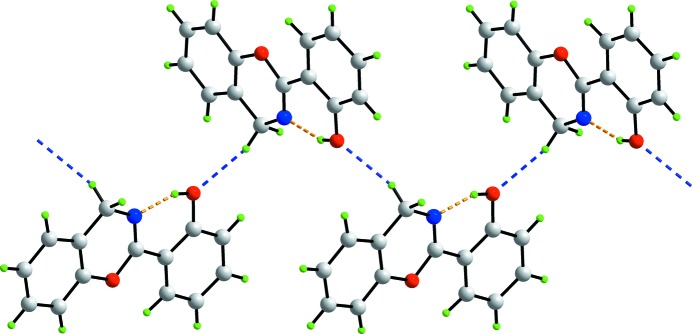
Supramolecular chain formation along the b axis mediated by C–H···O contacts (bue dashed lines) in the structure of (I).

## supplementary crystallographic information

### Comment

Reactions of various arene-aldehydes, ArCHO, *e.g.* Ar = Ph, thien-2-yl and pyridin2-yl, with ammonia, under basic conditions, have provided rac-2,4,5-Ar_3_-4,5-dihydro-1*H*-imidazoles, 1 (Hunter & Sims, 1972*a*, b; Corey, & Kühnle, 1997; Larter *et al.*, 1998; Chou *et al.*, 2004), see Fig. 3, either as the kinetic product, the *cis*-isomer, or the thermodynamic product, the *trans*-isomer. We reported similar products from these aldehydes using the mixture of NH_4_Cl and NEt_3_ as the ammonia source in MeOH (Fernandes *et al.*, 2007). In contrast, we have found that 2-hydroxybenzaldehyde, under these conditions produced (2: *R* = H).

The reaction of 2-hydroxybenzaldehyde with ammonia had previously been studied as a function of solvent (Kitan *et al.*, 1990). Thus in C_6_H_6_, hexane or Et_2_O, 2-HOC_6_H_4_CHNH was formed. In H_2_O, dioxan, MeOH or EtOH solution 2-HOC_6_H_4_CH(NCHC_6_H_4_OH-2)_2_ (3), was the product, while in MeOH with NH_4_OAc and NH_4_HCO_3_, both 3 and 4 were formed. Earlier, it had been reported that 2-hydroxybenzaldehyde, NH_4_OAc and HOAc in refluxing C_6_H_6_ produced 4 (Kanakarajan *et al.*, 1975). Interestingly, (2: *R* = H) has been shown to arise from the acid-catalysed decomposition of 4, alternatively obtained as the *cyclo*-condensation product of 2-hydroxybenzaldehyde with HONH_2_ (Meier *et al.*, 1979).

While the NMR spectra, in particular the two hydrogen singlet in the ^1^H NMR spectrum, suggested the compound (2: *R* = H) confirmation from the X-ray structure determination was considered to be of value, particularly since treatment of 6-nitro-2-hydroxybenzaldehyde with NH_4_OAc in acetic acid had been reported to give 5 (Beer *et al.*, 1948). However, no spectral details had been provided for 5. In the light of our findings, the structure of 5 could be modified to (2: *R* = NO_2_). The change from 5 to (2: *R* = NO_2_) merely involves a prototropic rearrangement. Herein, the crystal and molecular structure of (I) [= 2: *R* = H in Fig. 1] is described.

The molecular structure of (I), Fig. 2, is essentially planar with the RMS for the 17 non-hydrogen atoms being 0.035 Å. The maximum deviation is 0.049 (1) Å for the N1 atom. This conformation is stabilized by an intramolecular O–H···N hydrogen bond that completes an S(6) ring synthon. The most prominent interactions in the crystal structure are of the type C–H···O, Table 1, which lead to supramolecular chains along the *b* direction, Fig. 3.

### Experimental

A solution of 2-hydroxybenzaldehyde (3.7 g, 0.03 mol), ammonium chloride (3 g, 0.06 mol) and triethylamine (8.1 ml, 0.06 mol) in MeOH (30 ml) was refluxed for 8 h. The solvent was removed *in vacuo*, the residue was extracted into CHCl_3_, washed with water (2 *x* 1 5 ml), dried over magnesium chloride, and rotary evaporated. The pale coloured residue was recrystallized from EtOH to give colourless crystals. Anal. Calc. for C_14_H_11_NO_2_: C, 74.65; H, 4.92; N, 6.22. Found: C, 74.32; H, 4.80; N, 6.01%. ^1^H NMR (Me_2_CO-d6, 400 MHz): δ 13.02 (*s*, 1H), 7.96 (*dd*, J = 1.6, 8.0 Hz, 1H), 7.41–7.37 (*dt*, J = 1.7, 8.0 Hz, 1H), 7.33–7.28 (*m*, 1H), 7.20–7.14 (*m*, 3H), 6.91 (*m*, 2H), 4.82 (*s*, 2H) p.p.m. ^13^C NMR (CDCl_3_, 100 MHz): δ 134.1, 129.4, 128.1, 127.3, 126.3, 119.1, 118.0, 116.6, 44.1 p.p.m.; quaternary-C not detected.

### Refinement

The C-bound H atoms were geometrically placed (C–H = 0.95–0.99 Å) and refined as riding with *U_iso_*(H) = 1.*2U_eq_*(C). The hydroxyl-H was located from a difference map and refined with O–H = 0.840±0.001 Å, and with *U_iso_*(H) = 1.*5U_eq_*(O).

### Crystal data

**Table:**

C_14_H_11_NO_2_	*F*(000) = 472
*M**_r_* = 225.24	*D*_x_ = 1.424 Mg m^−^^3^
Monoclinic, *P*2/*n*	Mo *K*α radiation, λ = 0.71073 Å
Hall symbol: -P 2yac	Cell parameters from 10058 reflections
*a* = 14.1148 (8) Å	θ = 2.9–27.5°
*b* = 5.1725 (2) Å	µ = 0.10 mm^−^^1^
*c* = 15.8813 (8) Å	*T* = 120 K
β = 115.032 (2)°	Needle, orange
*V* = 1050.57 (9) Å^3^	0.55 × 0.08 × 0.08 mm
*Z* = 4	

### Data collection

**Table:**

Bruker–Nonius 95mm CCD camera on κ-goniostat diffractometer	1841 independent reflections
Radiation source: Bruker-Nonius FR591 rotating anode	1479 reflections with *I* > 2σ(*I*)
10 cm confocal mirrors	*R*_int_ = 0.066
Detector resolution: 9.091 pixels mm^-1^	θ_max_ = 25.0°, θ_min_ = 3.2°
φ and ω scans	*h* = −16→16
Absorption correction: multi-scan (*SADABS*; Sheldrick, 2003)	*k* = −6→6
*T*_min_ = 0.949, *T*_max_ = 0.992	*l* = −18→18
10487 measured reflections	

### Refinement

**Table:**

Refinement on *F*^2^	Secondary atom site location: difference Fourier map
Least-squares matrix: full	Hydrogen site location: inferred from neighbouring sites
*R*[*F*^2^ > 2σ(*F*^2^)] = 0.048	H-atom parameters constrained
*wR*(*F*^2^) = 0.122	*w* = 1/[σ^2^(*F*_o_^2^) + (0.0712*P*)^2^ + 0.1735*P*] where *P* = (*F*_o_^2^ + 2*F*_c_^2^)/3
*S* = 1.07	(Δ/σ)_max_ < 0.001
1841 reflections	Δρ_max_ = 0.32 e Å^−^^3^
158 parameters	Δρ_min_ = −0.32 e Å^−^^3^
1 restraint	Extinction correction: *SHELXL97* (Sheldrick, 2008), Fc^*^=kFc[1+0.001xFc^2^λ^3^/sin(2θ)]^-1/4^
Primary atom site location: structure-invariant direct methods	Extinction coefficient: 0.031 (5)

### Special details

**Table:**

Geometry. All s.u.'s (except the s.u. in the dihedral angle between two l.s. planes) are estimated using the full covariance matrix. The cell s.u.'s are taken into account individually in the estimation of s.u.'s in distances, angles and torsion angles; correlations between s.u.'s in cell parameters are only used when they are defined by crystal symmetry. An approximate (isotropic) treatment of cell s.u.'s is used for estimating s.u.'s involving l.s. planes.
Refinement. Refinement of *F*^2^ against ALL reflections. The weighted *R*-factor *wR* and goodness of fit *S* are based on *F*^2^, conventional *R*-factors *R* are based on *F*, with *F* set to zero for negative *F*^2^. The threshold expression of *F*^2^ > 2σ(*F*^2^) is used only for calculating *R*-factors(gt) *etc*. and is not relevant to the choice of reflections for refinement. *R*-factors based on *F*^2^ are statistically about twice as large as those based on *F*, and *R*- factors based on ALL data will be even larger.

### Fractional atomic coordinates and isotropic or equivalent isotropic displacement parameters (Å^2^)

**Table:**

	*x*	*y*	*z*	*U*_iso_*/*U*_eq_	
O1	0.87956 (9)	−0.1882 (2)	0.76593 (8)	0.0294 (3)	
H1O	0.8351	−0.0735	0.7382	0.044*	
O2	0.84545 (9)	0.3337 (2)	0.55121 (7)	0.0251 (3)	
N1	0.78226 (10)	0.1846 (2)	0.65809 (9)	0.0225 (3)	
C1	0.94605 (12)	−0.1824 (3)	0.72470 (10)	0.0224 (4)	
C2	1.02672 (13)	−0.3622 (3)	0.75286 (11)	0.0255 (4)	
H2	1.0334	−0.4852	0.7995	0.031*	
C3	1.09719 (13)	−0.3631 (3)	0.71350 (11)	0.0280 (4)	
H3	1.1518	−0.4875	0.7329	0.034*	
C4	1.08874 (13)	−0.1831 (3)	0.64558 (11)	0.0285 (4)	
H4	1.1375	−0.1839	0.6188	0.034*	
C5	1.00909 (12)	−0.0038 (3)	0.61741 (11)	0.0255 (4)	
H5	1.0039	0.1199	0.5714	0.031*	
C6	0.93574 (12)	−0.0004 (3)	0.65525 (10)	0.0210 (4)	
C7	0.84764 (12)	0.1812 (3)	0.62239 (10)	0.0214 (4)	
C8	0.76543 (12)	0.5157 (3)	0.51535 (10)	0.0218 (4)	
C9	0.76759 (13)	0.6767 (3)	0.44659 (11)	0.0253 (4)	
H9	0.8216	0.6614	0.4262	0.030*	
C10	0.68991 (13)	0.8601 (3)	0.40814 (11)	0.0271 (4)	
H10	0.6900	0.9711	0.3605	0.033*	
C11	0.61167 (13)	0.8827 (3)	0.43898 (11)	0.0277 (4)	
H11	0.5585	1.0094	0.4127	0.033*	
C12	0.61155 (12)	0.7197 (3)	0.50813 (11)	0.0257 (4)	
H12	0.5580	0.7361	0.5291	0.031*	
C13	0.68845 (12)	0.5327 (3)	0.54724 (10)	0.0222 (4)	
C14	0.69092 (13)	0.3531 (3)	0.62239 (11)	0.0252 (4)	
H14A	0.6898	0.4570	0.6743	0.030*	
H14B	0.6271	0.2450	0.5977	0.030*	

### Atomic displacement parameters (Å^2^)

**Table:**

	*U*^11^	*U*^22^	*U*^33^	*U*^12^	*U*^13^	*U*^23^
O1	0.0263 (7)	0.0350 (7)	0.0318 (7)	0.0037 (5)	0.0169 (6)	0.0083 (5)
O2	0.0243 (7)	0.0280 (6)	0.0264 (6)	0.0058 (5)	0.0141 (5)	0.0068 (4)
N1	0.0196 (7)	0.0265 (8)	0.0224 (7)	0.0004 (5)	0.0097 (6)	−0.0018 (5)
C1	0.0207 (9)	0.0248 (8)	0.0220 (8)	−0.0044 (6)	0.0093 (7)	−0.0042 (6)
C2	0.0253 (9)	0.0244 (8)	0.0241 (8)	−0.0006 (7)	0.0077 (7)	0.0015 (6)
C3	0.0234 (9)	0.0277 (9)	0.0305 (9)	0.0036 (7)	0.0090 (8)	−0.0017 (7)
C4	0.0244 (9)	0.0330 (10)	0.0318 (9)	0.0031 (7)	0.0157 (8)	−0.0004 (7)
C5	0.0252 (9)	0.0273 (9)	0.0260 (8)	−0.0004 (7)	0.0128 (7)	0.0008 (6)
C6	0.0182 (8)	0.0228 (8)	0.0200 (8)	−0.0023 (6)	0.0063 (7)	−0.0034 (6)
C7	0.0220 (9)	0.0216 (8)	0.0197 (8)	−0.0037 (6)	0.0079 (7)	−0.0024 (6)
C8	0.0191 (8)	0.0217 (8)	0.0219 (8)	−0.0001 (6)	0.0062 (7)	−0.0029 (6)
C9	0.0240 (9)	0.0277 (9)	0.0257 (8)	−0.0027 (7)	0.0120 (7)	−0.0016 (6)
C10	0.0298 (10)	0.0250 (9)	0.0239 (8)	−0.0014 (7)	0.0088 (7)	0.0016 (6)
C11	0.0265 (9)	0.0258 (9)	0.0265 (9)	0.0034 (7)	0.0070 (7)	−0.0011 (6)
C12	0.0222 (9)	0.0285 (9)	0.0256 (9)	0.0011 (7)	0.0094 (7)	−0.0042 (6)
C13	0.0217 (9)	0.0242 (8)	0.0190 (8)	−0.0022 (6)	0.0069 (7)	−0.0050 (6)
C14	0.0233 (9)	0.0297 (9)	0.0257 (8)	0.0018 (7)	0.0133 (7)	0.0000 (6)

### Geometric parameters (Å, °)

**Table:**

O1—C1	1.3530 (19)	C5—H5	0.9500
O1—H1O	0.8403	C6—C7	1.467 (2)
O2—C7	1.3680 (18)	C8—C13	1.382 (2)
O2—C8	1.3937 (19)	C8—C9	1.384 (2)
N1—C7	1.271 (2)	C9—C10	1.381 (2)
N1—C14	1.458 (2)	C9—H9	0.9500
C1—C2	1.389 (2)	C10—C11	1.390 (2)
C1—C6	1.410 (2)	C10—H10	0.9500
C2—C3	1.381 (2)	C11—C12	1.385 (2)
C2—H2	0.9500	C11—H11	0.9500
C3—C4	1.391 (2)	C12—C13	1.389 (2)
C3—H3	0.9500	C12—H12	0.9500
C4—C5	1.378 (2)	C13—C14	1.501 (2)
C4—H4	0.9500	C14—H14A	0.9900
C5—C6	1.400 (2)	C14—H14B	0.9900
			
C1—O1—H1O	104.4	C13—C8—C9	122.28 (15)
C7—O2—C8	117.29 (12)	C13—C8—O2	121.33 (13)
C7—N1—C14	121.61 (13)	C9—C8—O2	116.39 (13)
O1—C1—C2	118.12 (14)	C10—C9—C8	118.93 (15)
O1—C1—C6	122.02 (14)	C10—C9—H9	120.5
C2—C1—C6	119.86 (14)	C8—C9—H9	120.5
C3—C2—C1	120.39 (15)	C9—C10—C11	120.16 (14)
C3—C2—H2	119.8	C9—C10—H10	119.9
C1—C2—H2	119.8	C11—C10—H10	119.9
C2—C3—C4	120.46 (15)	C12—C11—C10	119.75 (15)
C2—C3—H3	119.8	C12—C11—H11	120.1
C4—C3—H3	119.8	C10—C11—H11	120.1
C5—C4—C3	119.52 (15)	C11—C12—C13	121.03 (15)
C5—C4—H4	120.2	C11—C12—H12	119.5
C3—C4—H4	120.2	C13—C12—H12	119.5
C4—C5—C6	121.26 (14)	C8—C13—C12	117.85 (14)
C4—C5—H5	119.4	C8—C13—C14	119.64 (14)
C6—C5—H5	119.4	C12—C13—C14	122.51 (14)
C5—C6—C1	118.50 (14)	N1—C14—C13	113.49 (13)
C5—C6—C7	121.67 (13)	N1—C14—H14A	108.9
C1—C6—C7	119.81 (14)	C13—C14—H14A	108.9
N1—C7—O2	126.42 (14)	N1—C14—H14B	108.9
N1—C7—C6	121.08 (13)	C13—C14—H14B	108.9
O2—C7—C6	112.49 (13)	H14A—C14—H14B	107.7
			
O1—C1—C2—C3	−179.47 (14)	C1—C6—C7—O2	−176.25 (13)
C6—C1—C2—C3	0.3 (2)	C7—O2—C8—C13	−3.2 (2)
C1—C2—C3—C4	0.4 (2)	C7—O2—C8—C9	176.88 (13)
C2—C3—C4—C5	−0.2 (3)	C13—C8—C9—C10	−0.3 (2)
C3—C4—C5—C6	−0.6 (2)	O2—C8—C9—C10	179.67 (13)
C4—C5—C6—C1	1.2 (2)	C8—C9—C10—C11	0.5 (2)
C4—C5—C6—C7	−176.86 (14)	C9—C10—C11—C12	−0.3 (2)
O1—C1—C6—C5	178.68 (13)	C10—C11—C12—C13	−0.2 (2)
C2—C1—C6—C5	−1.0 (2)	C9—C8—C13—C12	−0.2 (2)
O1—C1—C6—C7	−3.2 (2)	O2—C8—C13—C12	179.85 (13)
C2—C1—C6—C7	177.07 (13)	C9—C8—C13—C14	−179.68 (14)
C14—N1—C7—O2	2.7 (2)	O2—C8—C13—C14	0.4 (2)
C14—N1—C7—C6	−176.26 (13)	C11—C12—C13—C8	0.4 (2)
C8—O2—C7—N1	1.7 (2)	C11—C12—C13—C14	179.89 (15)
C8—O2—C7—C6	−179.26 (11)	C7—N1—C14—C13	−5.1 (2)
C5—C6—C7—N1	−179.12 (14)	C8—C13—C14—N1	3.6 (2)
C1—C6—C7—N1	2.8 (2)	C12—C13—C14—N1	−175.88 (13)
C5—C6—C7—O2	1.8 (2)		

### Hydrogen-bond geometry (Å, °)

**Table:**

*D*—H···*A*	*D*—H	H···*A*	*D*···*A*	*D*—H···*A*
O1—H1O···N1	0.84	1.78	2.5586 (16)	154
C14—H14A···O1^i^	0.99	2.45	3.3551 (19)	151

Symmetry codes: (i) −*x*+3/2, *y*+1, −*z*+3/2.
